# Portal Venous Thrombosis after Percutaneous Cryoablation for Renal Cell Carcinoma

**DOI:** 10.15586/jkcvhl.v11i2.347

**Published:** 2024-07-15

**Authors:** Takahiro Kawabata, Toshihiro Iguchi, Yusuke Matsui, Koji Tomita, Mayu Uka, Noriyuki Umakoshi, Takao Hiraki

**Affiliations:** 1 Department of Radiology, Okayama University Hospital, Okayama, Japan;; 2 Department of Radiological Technology, Faculty of Health Sciences, Okayama University, Okayama, Japan;; 3 Department of Radiology, Faculty of Medicine, Dentistry and Pharmaceutical Sciences, Okayama University, Okayama, Japan

**Keywords:** Complication, Cryoablation, Portal vein, Renal cell carcinoma, Thrombus

## Abstract

A 50-year-old man with von Hippel–Lindau disease underwent cryoablation (CRA) for two adjacent renal cell carcinomas in the upper pole of his right kidney. Although computed tomography (CT) immediately after CRA revealed involvement of part of the liver parenchyma in the ice-ball, the treatment was completed without complications. Contrast-enhanced CT on day 2 post-CRA revealed a thrombus in the portal vein of segment 6 near the ablated liver parenchyma, prompting the initiation of oral anticoagulation. The patient was discharged on day 4 after CRA without any sequelae, and a follow-up contrast-enhanced CT done 6 weeks later demonstrated resolution of the portal vein thrombus.

## Introduction

Treatment with percutaneous cryoablation (CRA) for small renal cell carcinoma (RCC) has been increasing due to its suitability for patients ineligible for surgery, less invasiveness, preservation of renal function, and high safety and efficacy. Current guidelines recommend thermal ablation therapy, including CRA, as the primary treatment for patients with small and localized RCC (i.e., T1a RCC) (1, 2). With percutaneous CRA, major complications are usually rare (reported rate, 0–7.2%), mortality is very low (reported rate, 0–1.6%), and most complications are minor (i.e., grade 1 bleeding) and do not pose a clinical problem (3). Thrombotic complications are also rare (reported rate, 0–3.5%) (3), but portal vein thrombosis is poorly understood. Therefore, we describe a case of portal vein thrombus formation post-CRA for RCC involving an ice-ball near the liver parenchyma.

## Case Report

A 50-year-old man with von Hippel–Lindau disease, who had previously received treatment for multiple RCCs, was admitted for percutaneous CRA to address two new RCCs (10 × 10 mm and 11 × 9 mm in diameter, respectively) in the right upper pole of the kidney. The results of the initial blood test were as follows: aspartate transaminase (AST) at 15 U/L, alanine transaminase (ALT) at 17 U/L, lactate dehydrogenase at 187 U/L, total bilirubin at 0.70 mg/dL, creatine at 1.39 mg/dL, and estimated glomerular filtration rate at 44.0 mL/min/1.73 m^2^. The patient did not have any risk factors for thrombosis (e.g. diseases causing hypercoagulability, history of venous thrombosis, obesity, smoking, and diabetes mellitus) other than RCC.

The day before the CRA, renal arterial embolization was performed on the target RCCs using iodized oil (Lipiodol Ultra Fluid; Guerbet Japan, Tokyo, Japan) and a gelatin sponge (Gelpart, Nippon Kayaku, Tokyo, Japan) to enhance tumor ischemia, reduce the potential risk of procedural bleeding and dissemination, and improve tumor localization on computed tomography (CT) images. The CRA was performed using an argon-helium-based device (VISUAL ICE, Boston Scientific, Marlborough, MA, USA) and two 17-gauge cryoprobes (IceSphere; Boston Scientific) under local anesthesia and CT fluoroscopy guidance. The two adjacent RCCs were ablated simultaneously in two freezing cycles (10 and 12 min), with 2 min of passive thawing in between. After each cycle, the operator confirmed that the target was within a low-attenuation area, known as an ice-ball, on CT scan with sufficient circumferential ablation margin. Some of the liver parenchyma was included in the ice-ball, but the procedure was carried out without any immediate complications.

**Figure 1:**
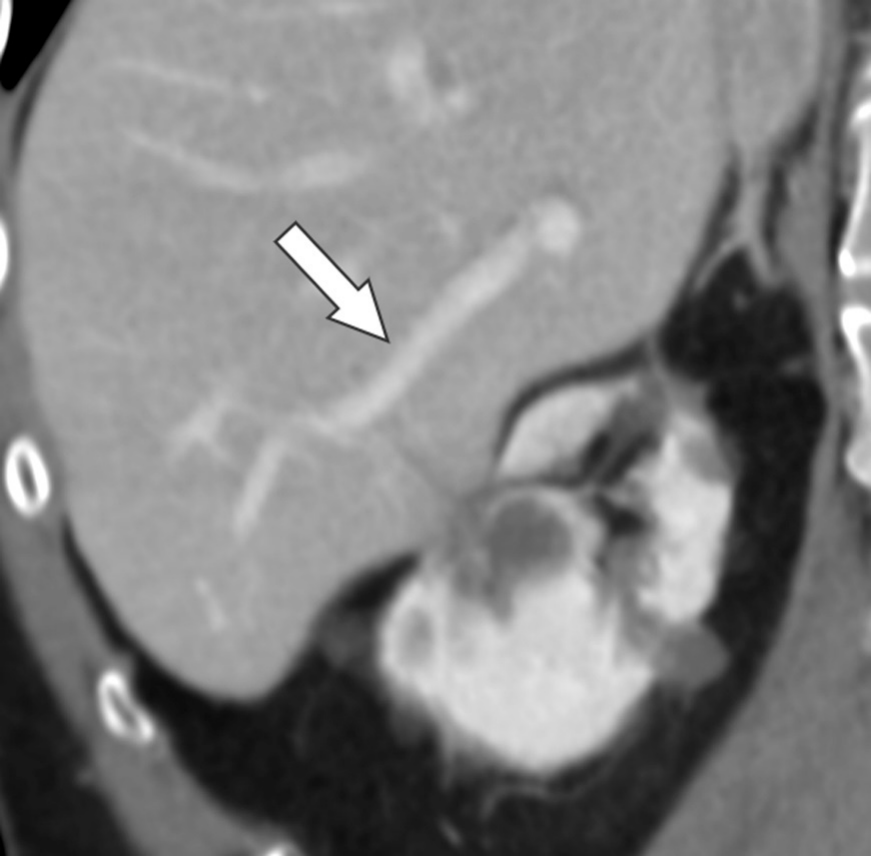
Oblique coronal contrast-enhanced CT before cryoablation reveals the normal liver parenchyma and patent portal vein branch (arrow).

**Figure 2:**
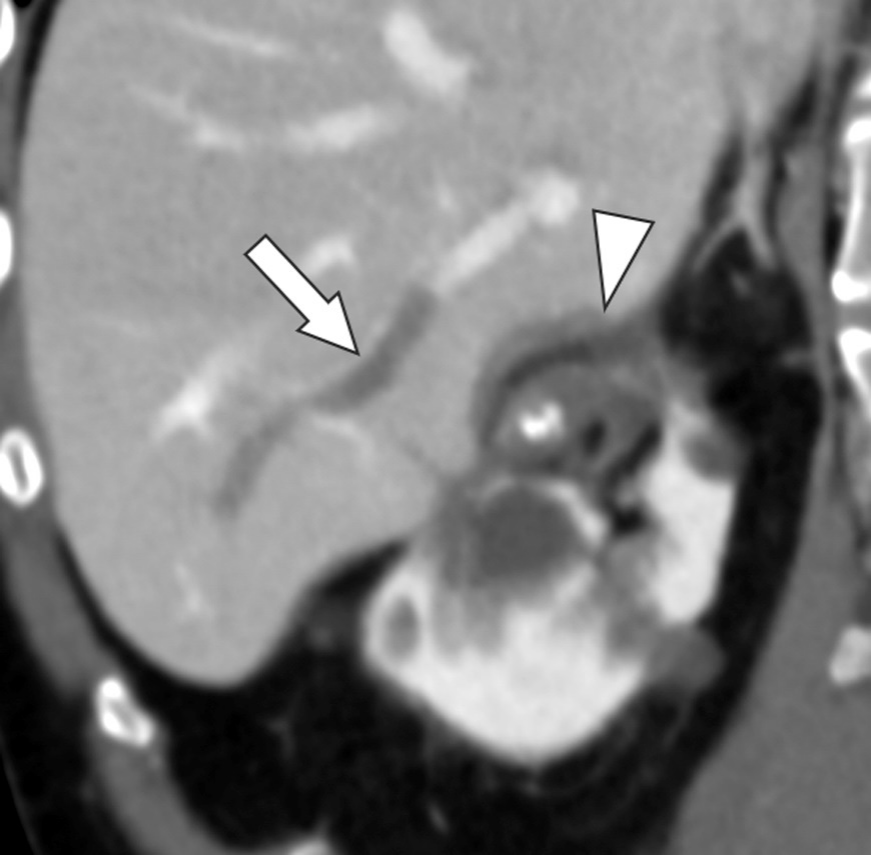
Oblique coronal contrast-enhanced CT 2 days after cryoablation reveals the ablated liver parenchyma (arrowhead) and portal venous thrombus (arrow).

On day 2 post-CRA, contrast-enhanced CT revealed a thrombus in the portal vein in segment 6 near the ablated liver parenchyma (Figures 1 and 2), and oral anticoagulant therapy (edoxaban, 60 mg/day) was initiated. The patient had no specific symptoms, and his laboratory results were as follows: AST 27 U/L, ALT 46 U/L, lactate dehydrogenase 243 U/L, total bilirubin 0.53 mg/dL, creatine 1.31 mg/dL, and estimated glomerular filtration rate 47.0 mL/min/1.73 m^2^. The patient was discharged on day 4 post-CRA without any sequelae, and a follow-up contrast-enhanced CT performed 6 weeks later revealed the resolution of the portal vein thrombus.

The approval of our institutional review board is not required for a case report. Written informed consent was obtained from the patient prior to CRA and imaging examinations.

## Discussion

With percutaneous CRA, complications occur in 3.2–30.3% of patients (3). The most prevalent complications include bleeding (e.g., hematoma and hematuria), ureteral strictures, pneumothorax, bowel injury, nerve injury, and thrombosis (3).

Renal CRA–related thrombotic complications, including pulmonary arterial embolism and venous thrombus, have been reported at a frequency of 0–3.5% (3). In one study using rat liver, the inflammatory and coagulation response was more intense with CRA than with other ablative methods such as radiofrequency or laser ablation (4). Thromboses are typically managed with medication and are not severe when diagnosed and treated properly. However, it is crucial for operators to recognize that this complication, although rare, can be dangerous, with one reported fatality (5). Performing imaging evaluation of post-CRA thrombus in all patients may be excessive due to its low incidence, except for certain patients such as those with longer procedure time, risk factors for thrombosis, or very high D-dimer levels after CRA. We could detect portal thrombus by a routine abdominal dynamic CT after CRA, and no special imaging examinations were performed.

**Figure 3:**
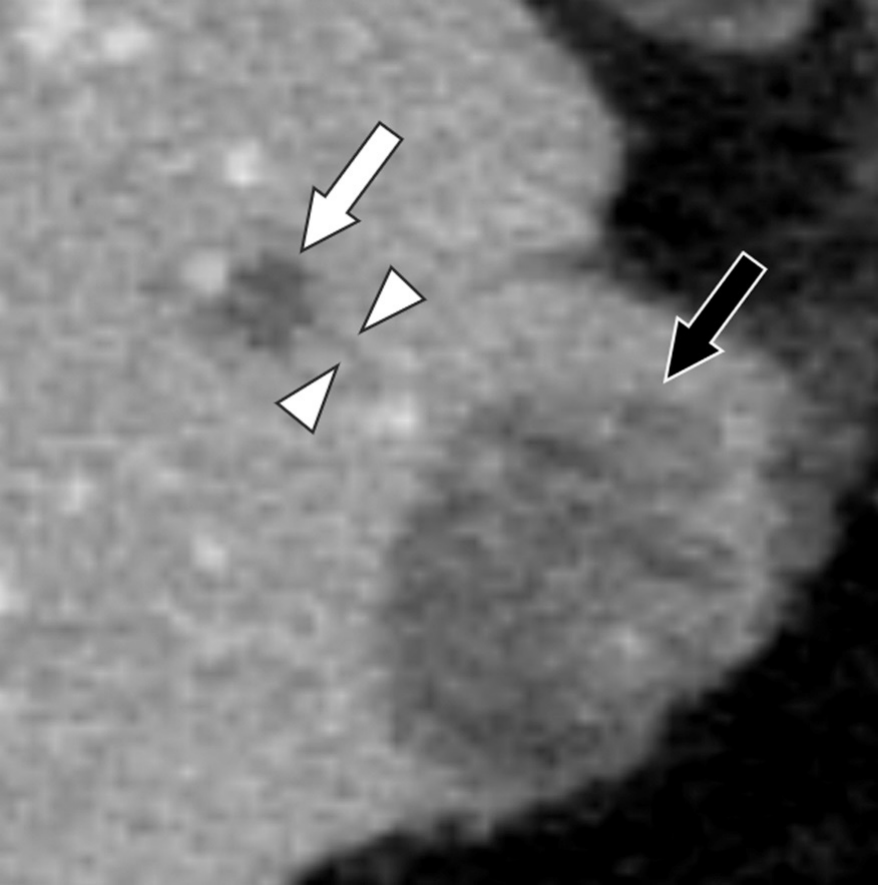
Axial contrast-enhanced CT 2 days after cryoablation reveals a small portal venous thrombus (arrowheads) adjacent to the subsegmental portal venous thrombus (white arrow) toward the ablation zone. There is no continuity between the small thrombus and the ablation zone (black arrow).

In percutaneous CRA of 223 liver tumors in 135 patients, Sainani et al. reported that the incidence of portal and hepatic venous thrombosis after ablation was 24% (54/233): these included 49 thromboses in portal vein branches, 4 in branches of both portal and hepatic veins, and 1 in a hepatic vein branch (6). The thrombosed veins were outside but adjacent to the ablation zone in 36 patients (66.7%) and within the ablation zone in 18 patients (33.3%) (6). They speculated that one mechanism of thrombus formation outside the ablation zone is thrombosis resulting from freezing within the ice-ball but outside the lethal isotherm, citing the results of Littrup et al. (7). In our patient, no portal venous thrombus was in contact with the ablation zone; however, there was a small portal vein thrombus near the ablation zone (Figure 3). There may have been a subvisible thrombus contiguous with the ablation zone.

Although portal venous thrombosis may develop during renal CRA when the liver parenchyma is included in the ablation zone, the incidence and risk factors of portal venous thrombosis post-CRA for RCC are unknown. In the CRA for liver tumors, no differences were observed in age, sex, tumor characteristics, or procedural parameters between patients who developed thrombosis and those who did not (6). A thrombosis is difficult to detect using laboratory investigations after ablation. Changes in liver enzymes (e.g., elevated AST and ALT) are unlikely to help diagnose portal venous thrombus formation because they can be attributed to necrosis of the tumor, renal parenchyma (8), and liver parenchyma involved in the ice-ball. Contrast-enhanced magnetic resonance imaging (MRI) has been reported to be more useful (9), but it is difficult to employ in all patients, whereas color Doppler ultrasound may be useful and more convenient.

## Conclusion

Many operators would not be concerned about post-ablative portal venous thrombus if the liver was not ablated directly during renal CRA. However, we recommend consideration of portal venous thrombus formation as a potential complication of renal CRA if the ice-ball involves the liver parenchyma.
